# Investigation of the role of X chromosome inactivation and androgen receptor CAG repeat polymorphisms in patients with recurrent pregnancy loss: a prospective case–control study

**DOI:** 10.1186/s12884-022-05113-z

**Published:** 2022-11-02

**Authors:** Yilun Sui, Jing Fu, Shuo Zhang, Lu Li, Xiaoxi Sun

**Affiliations:** 1 Shanghai Ji Ai Genetics and IVF Institute, Obstetrics and Gynecology Hospital, Fudan University, Shanghai, People’s Republic of China; 2 Key Laboratory of Female Reproductive Endocrine Related Diseases, Obstetrics and Gynecology Hospital, Fudan University, Shanghai, People’s Republic of China

**Keywords:** Recurrent pregnancy loss, X-chromosome inactivation, Androgen receptor CAG repeat polymorphisms, Preimplantation embryo aneuploidy, Ovarian reserve

## Abstract

**Background:**

Previous research has revealed that skewed X chromosome inactivation (SXCI) and androgen receptor (AR) CAG polymorphisms are associated with increased risk of recurrent pregnancy loss (RPL); however, the results are conflicting, and the underlying mechanisms remain unclear. This study investigated the role of SXCI and AR CAG polymorphisms in patients with RPL and explored whether the underlying mechanisms were related to the ovarian reserve and preimplantation embryo aneuploidy.

**Methods:**

This was a prospective case-control study carried out in a tertiary hospital-based reproductive medicine center. An external validation RPL cohort was recruited during the study period. Data on baseline and cycle characteristics were collected. X-chromosome inactivation (XCI) was measured using a human AR assay. AR polymorphisms were assessed using quantitative fluorescent polymerase chain reactions and direct sequencing. Blastocysts of the patients with RPL were tested by single nucleotide polymorphism microarray based preimplantation genetic testing for aneuploidy.

**Results:**

In total, 131 patients with idiopathic RPL and 126 controls were included for the case-control study. Patients with RPL exhibited a significantly more skewed XCI distribution pattern (67.71 ± 10.50 vs. 64.22 ± 10.62, p = 0.011), as well as significantly shorter bi-allelic mean (18.56 ± 1.97 vs. 19.34 ± 2.38, p = 0.005) and X-weighted bi-allelic mean (18.46 ± 2.02 vs. 19.38 ± 2.53, p = 0.001) of AR CAG repeats. Multivariate logistic regression models indicated that CAG repeat < 20, SXCI, and duration of stimulation were independently associated with the risk of RPL. However, SXCI and AR CAG polymorphisms were not associated with ovarian reserve or preimplantation embryo aneuploidy in the RPL group, and the same results were attained in a separate validation cohort of 363 patients with RPL.

**Conclusion:**

SXCI and AR CAG polymorphisms are related to RPL; however, these two factors do not lead to RPL by affecting the ovarian reserve or increasing embryo aneuploidy. The roles of SXCI and AR CAG in RPL may involve other mechanisms that require further investigation.

**Trial registration:**

NCT02504281, https://www.clinicaltrials.gov (Date of registration, 21/07/2015; date of enrolment of the first subject, 30/07/2015).

## Background

The diagnostic criteria for recurrent pregnancy loss (RPL) vary between three or more consecutive pregnancy losses [1, 2] and two or more pregnancy losses confirmed using ultrasonography or histology [3, 4]. According to the definition of European Society of Human Reproduction and Embryology (ESHRE), a diagnosis of RPL could be considered after the loss of two or more pregnancies before 24 weeks of gestation, including non-visible pregnancy losses (biochemical pregnancy losses and/or resolved and treated pregnancies of unknown location) [5]. RPL is experienced by approximately 1−5% of women trying to conceive, and can be the result of chromosomal abnormality, uterine anatomical defects, autoimmune disorders, and endometrial dysfunction. However, the etiology remains unknown in approximately 50% of all RPL cases, and limited evidence-based therapies exist, posing challenges to both physicians and patients. Studies have reported that patients with RPL tend to produce aneuploid embryos [6, 7], which are associated with 50−60% of identifiable causes of RPL. Therefore, preimplantation genetic testing for aneuploidy (PGT-A) can improve live birth rates in patients with RPL undergoing frozen embryo transfer [8].

X-chromosome inactivation (XCI) is a unique biological phenomenon observed in women. The process occurs during early embryonic development [9–11] when a maternally or paternally derived X chromosome (Xm or Xp) is randomly inactivated to attain dosage compensation in women. Theoretically, the random process leads to a normal distribution of the XCI skew in the female population. Non-random inactivation results in an individual with most or even all her somatic cells having the same active Xm or Xp, which is known as skewed XCI (SXCI). SXCI is a major cause of discontinuity of dominance and recessiveness, as well as penetrance and expressivity of X-linked traits.^12^ In heterozygous females with SXCI, the X-linked transcriptional and allelic dosages of silenced genes are unbalanced and may be functionally homozygous [12]. SXCI occurs in 2.7−3.5% of the normal population and its prevalence is significantly higher in patients with RPL (approximately 9.9%), ovarian dysfunction (approximately 15%), autoimmune diseases (approximately 6–30%), breast cancer (approximately 11.2%), and other hormone-sensitive diseases [13–18].

The androgen receptor (AR) gene is located at Xq11-12 [19] and has a polymorphic trinucleotide CAG repeat in exon 1 that encodes the polyglutamine tract in the N-terminal transactivating domain [20]. An inverse correlation between the length of CAG repeats and AR transcriptional activity has been demonstrated in vitro [21, 22]. Furthermore, clinical investigations suggest that CAG polymorphisms are associated with disorders sensitive to androgens or estrogens such as polycystic ovary syndrome [23] and breast cancer [24], and may affect ovarian function and folliculogenesis [25, 26].

Previous research has shown that SXCI and AR CAG polymorphisms are associated with increased risk of RPL; however, the results are conflicting, and the underlying mechanisms remain unclear. The results of our previous meta-analysis demonstrated that extreme skewing of SXCI (≥ 90%) is associated with idiopathic RPL with ≥ 3 pregnancy losses, while the association was not significant when RPL was defined as ≥ 2 losses or SXCI was defined as ≥ 85% [13]. The study by Aruna et al. reported significantly longer AR CAG repeats in women with RPL than in healthy controls [27], while the results of Chuan et al. demonstrated that significantly shorter CAG repeat lengths were associated with an increased risk of RPL [28]. Besides the conflicting results, none of the existing research explains this relationship between SXCI and AR CAG polymorphisms with RPL. Blyth et al. identified three studies with consistent results showing that SXCI was more common in women with RPL secondary to embryo aneuploidy in their systematic review [29–32]. However, previous studies on SXCI and aneuploidy were limited in sample size, and they investigated the karyotypes of products of conception from pregnancy loss, whereas PGT-A has enabled the analysis of the chromosome status of preimplantation embryos so that we can better understand the mechanisms underlying the patients’ tendency toward RPL. Since SXCI and AR CAG polymorphisms are variations involving the X chromosome, which represents approximately 5% of the haploid human genome and is enriched for sex-related genes regulating sexual development and ovarian function, we postulated that SXCI and AR CAG polymorphisms may affect ovarian function, follicle development, and embryo aneuploidy through intricate genomic interaction networks, thereby increasing the risk of RPL. Therefore, the present study was designed to investigate the role of XCI and AR CAG polymorphisms in patients with RPL and explore whether the underlying mechanisms are related to the ovarian reserve and preimplantation embryo aneuploidy.

## Methods

### Study design

This study consisted of two parts to investigate the role of SXCI and AR CAG polymorphisms in recurrent pregnancy loss. Part one was a prospective case–control study with a 1:1 ratio to compare the SXCI status and AR CAG polymorphisms in patients with RPL and healthy controls, and to investigate the associations of SXCI and AR CAG polymorphisms with ovarian reserve or blastocyst aneuploidy in the RPL group. Part two was an external validation cohort study recruiting patients with RPL to confirm the associations revealed in part one with a sufficient sample size.

### Participants

We recruited patients aged 18 − 43 years who visited the Shanghai Ji-Ai Genetics and IVF Institute between July 2015 and June 2021. Patients who sought PGT-A following ≥ 3 pregnancy losses, including biochemical pregnancies [5], were included in the RPL group (from July 2015 to December 2016) or the RPL validation cohort (from Jan 2017 to June 2021). The control group included women with no history of spontaneous pregnancy loss who sought intracytoplasmic sperm injection (ICSI) treatment due to only male factors. Considering heterogeneous ethics background might be a confounding factor for genetic polymorphisms, only Chinese Han population were enrolled. Each participant was required to have regular menstrual cycles of 24 − 38 days, a body mass index (BMI) of 18.5–24.9 kg/m^2^, and the 46, XX karyotype for the participant and 46, XY for the partner. All patients with RPL were negative for anti-phospholipid antibody and antinuclear antibody. The exclusion criteria for the participants were as follows: (1) history of any other endocrine disorder, such as polycystic ovary syndrome or abnormal thyroid stimulating hormone, free T3, or free T4 levels; (2) history of ovarian surgery or endometriosis; (3) history of autoimmune diseases, diagnosed thrombophilia (such as Factor V-Leiden or prothrombin G20210A mutation) or uterine abnormalities (such as adenomyosis, submucous myoma, non-submucous myoma > 4 cm and/or with compressed endometrium or uterine cavity lesions); (4) history of smoking, radio- or chemotherapy; and (5) the male partner having severe oligozoospermia, asthenospermia, or teratospermia.

The study protocol was approved by the research ethics committee of Shanghai Ji-Ai Genetics and IVF Institute (JIAI E2015-02, NCT02504281, www.clinicaltrials.gov). All participants provided written informed consent.

### Clinical and biochemical measurements

Data on clinical characteristics, including baseline and stimulation cycle parameters, were collected. Peripheral blood was sampled on day 2 − 3 of the menstrual cycle in each participant to determine the basal sex hormone concentrations using a radioimmunoassay. Ovarian function measurements included antral follicle count (AFC), and the anti-Müllerian hormone (AMH) and basal follicle-stimulating hormone (FSH) levels. Participants were identified as having diminished ovarian reserve (DOR) when the AMH level was < 1.1 ng/mL or AFC was ≤ 7 [33, 34].

### SXCI and AR CAG assessment

#### DNA extraction

Genomic DNA was extracted from peripheral blood leukocytes of every participant using the NucleoSpin Dx Blood Kit (Macherey-Nagel, Duren, Germany) in accordance with the manufacturer’s instructions. The DNA concentration and integrity were determined using a NanoDrop 2000 spectrophotometer (Thermo Fisher Scientific, Waltham, MA, USA).

#### X chromosome inactivation analysis

The XCI patterns were assessed based on the allele-specific DNA methylation of the AR exon 1 CAG repeat (human androgen receptor assay [HUMARA]), which is the gold standard for XCI analysis [35]. Polymorphic microsatellites with various numbers of (CAG)n of the AR gene can be used to identify different alleles on X chromosomes. Genomic DNA was digested with HpaII (Roche Diagnostic Systems, Penzberg, Germany) according to the manufacturer’s instructions. Hpall digests only unmethylated (active) DNA segments, leaving the undigested methylated (inactive) DNA template intact for amplification. Digested and mock-digested genomic DNA from the same woman were amplified using PCR with primers, as previously described [36]. The forward primer was 5(6)-carboxyfluorescein-labelled. The thermal cycling conditions were as follows: 94 °C for 2 min (one cycle), 98 °C for 10 s, 60 ℃ for 30 s, and 68 ℃ for 10 s (30 cycles), followed by 68 ℃ for 2 min, and 16 ℃ for 1 min (one cycle). Microsatellite fragment analysis was performed using an ABI3730 DNA Analyzer (Applied Biosystems, Foster City, CA, USA). The allele sizes, number of CAG repeats, and peak areas were analyzed using the Peak Scanner Software version 3.0 (Applied Biosystems).

DNA from a healthy man was used as a control in each run of the assay because the male X chromosome is always unmethylated. Samples homozygous for the AR (CAG)n gene locus were excluded from the XCI analysis due to the inability to distinguish between the two alleles. The degree of XCI was calculated for the heterozygous samples according to a previously published protocol [37]. Both 85% (highly skewed) and 90% (extremely skewed) inactivations of a particular X chromosome were used as the cut-off points for SXCI [13].

#### Androgen receptor (CAG)n repeat polymorphism analysis

The number of CAG repeats was calculated relative to a series of standards obtained using direct sequencing. The AR (CAG)n repeat polymorphism profiles were analyzed in three modes [38] as follows: (1) two independent values that represented both CAG repeat alleles; (2) the mean value of the two alleles (biallelic mean, BAM); and (3) X-weighted-biallelic-mean (XWBM), calculated by averaging the (CAG)n of each allele multiplied by its percentage of activation.

### Ovarian stimulation, embryo culture, and PGT-A

An antagonist protocol for controlled ovarian hyperstimulation was used for each participant. Treatment with recombinant human FSH (Gonal-f; Merck Serono, Geneva, Switzerland) was initiated on the 2nd or 3rd day of the menstrual cycle with a starting dose of 150 − 300 IU/day adjusted for age, BMI, AFC, FSH, and AMH levels. Gonadotropin-releasing hormone antagonist (Cetrotide; Merck Serono) was administered at a dose of 0.25 mg/day when the dominant follicle reached 14 mm in size or the serum E2 level reached 350 pg/mL. This treatment continued until a leading follicle reached 18 mm or two follicles reached 16 mm in size. Subsequently, 5,000 − 10,000 IU of human chorionic gonadotropin (Livzon, Zhuhai, China) was administered as a trigger and oocytes were retrieved 36 h later. ICSI and blastocyst culture were performed for all participants in accordance with IVF laboratory guidelines, and single nucleotide polymorphism microarray based PGT-A was administered to the patients with RPL as per the manufacturer’s instructions (Infinium HD Assay Ultra Protocol Guide, Illumina Inc., San Diego, CA, USA). Mosaicism calls were made when 20–80% of the biopsied cells were aneuploid.

### Statistical analysis

For the case–control study, the median CAG repeat number of the control group [36] was used as the cut-off for CAG polymorphisms. As the estimated distribution of < AR CAG cut-off in the control group was 50%, and an odds ratio (OR) between < AR cut-off and risk of RPL was supposed to be 2.5 [28], a minimum of 104 patients per group had to be included in the case–control study to detect such a difference with 90% statistical power and a two-sided 0.05 level of significance, as was calculated by PASS2021 software. For the validation cohort study, the sample size relied on an Events Per Variable criterion (EPV ≥ 10) for the binary logistic regression. As was revealed in the case–control study, the incidence of DOR in patients with RPL was approximately 20%, the number of confounders adjusted in the logistic regression model was 7, and a minimum of 350 patients with RPL had to be included.

Values are presented as average ± standard deviation for continuous data and were compared using Student’s t-test or Mann − Whitney U-test. The chi-square and Fisher’s exact tests were applied as appropriate for categorical variables. Departures from Hardy-Weinberg equilibrium (HWE) were tested to determine whether the frequencies of AR CAG repeat polymorphisms were consistent with the genetic balance. The adjusted ORs and 95% confidence intervals (95% CIs) of SXCI and AR CAG polymorphisms associated with RPL risk were examined by multivariate logistic regression analyses. The relationships of SXCI and AR CAG polymorphisms with ovarian reserve or blastocyst euploidy were evaluated using logistic regressions (Backward LR) that were adjusted for age, AFC, AMH, and other possible confounders. Data were analyzed using the Statistical Package for the Social Sciences (SPSS) software (version 20.0, SPSS, Inc., Chicago, IL, USA). The statistical significance level for all tests was set at P < 0.05.

## Results

### Case–control study

#### Baseline and cycle characteristics of patients in the RPL and control group

**Fig. 1:**
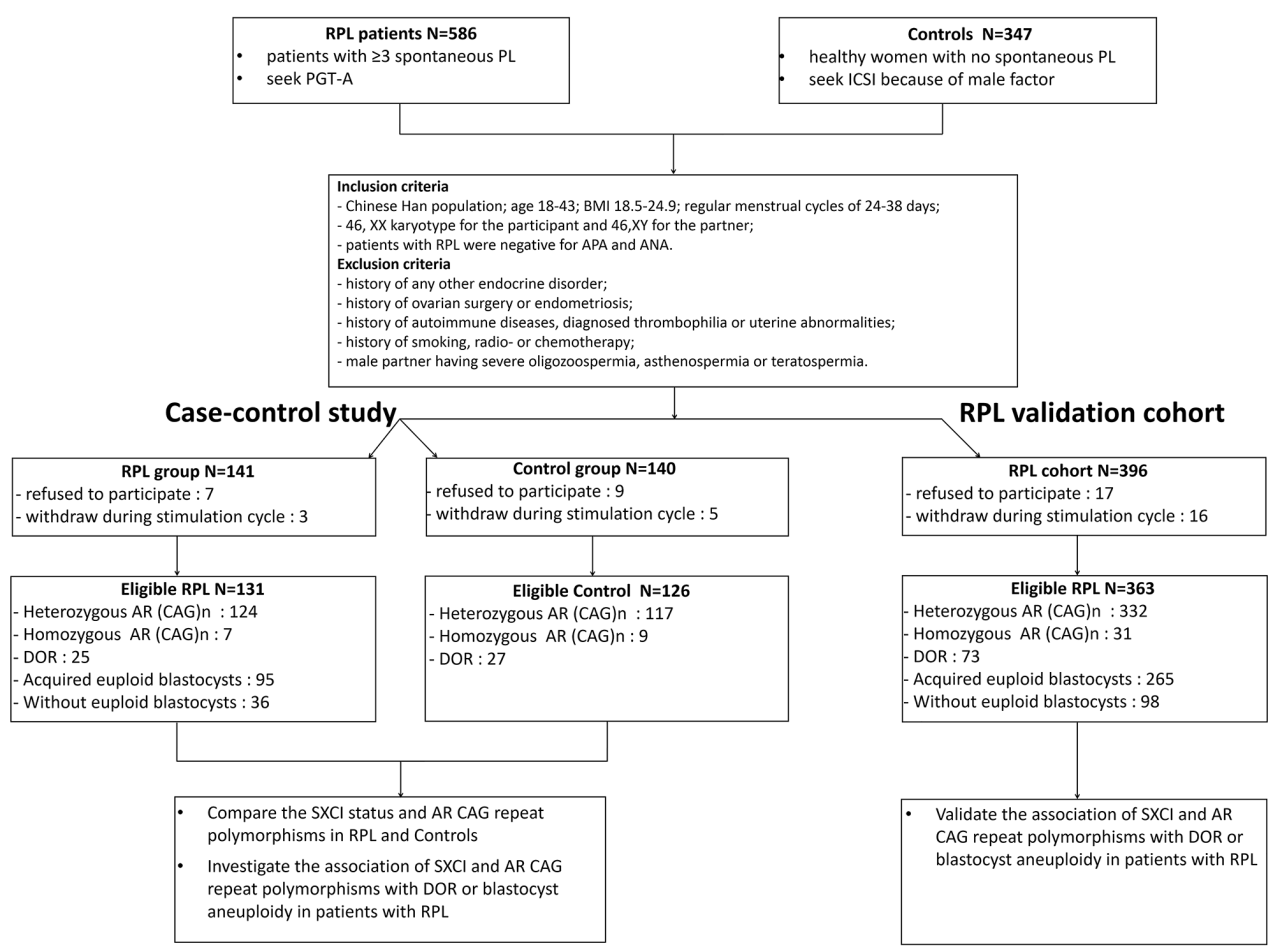
Flowchart of the study. PL = pregnancy loss; PGT-A = preimplantation genetic testing for aneuploidy; ICSI = intracytoplasmic sperm injection; AR = androgen receptor; DOR = diminished ovarian reserve

The case–control study included 131 patients with RPL and 126 controls (Fig. 1). The baseline characteristics and ovarian reserve parameters did not differ significantly between the groups (Table 1). The mean duration of ovarian stimulation was 9.39 ± 2.50 days and 10.41 ± 3.03 days in the RPL and control groups, respectively (p = 0.003), whereas other variables of ovarian stimulation did not differ significantly between the two groups, indicating faster follicle development in the RPL group.

**Table 1:** Baseline and cycle characteristics of the women in the RPL and control groups

	RPL group	Control group	P-value
	**N = 131**	** N = 126**	
Age (years)	33.12 ± 4.75	33.00 ± 6.00	0.856
BMI	22.16 ± 2.79	21.73 ± 3.10	0.126
No. of pregnancy losses	3.14 ± 0.81	0.31 ± 0.67	**0.000**
E2 (pg/ml)	40.59 ± 19.76	39.50 ± 17.58	0.644
T (ng/dL)	43.71 ± 23.52	44.89 ± 24.85	0.694
**Ovarian reserve**			
AMH	4.10 ± 3.23	3.95 ± 3.07	0.713
FSH on day 2	7.78 ± 2.60	7.97 ± 2.68	0.566
AFC	10.39 ± 5.44	11.46 ± 6.24	0.143
**Cycle and embryo characteristics**			
Duration of stimulation	9.39 ± 2.50	10.41 ± 3.03	**0.003**
Total dosage of gonadotropins	26.43 ± 11.61	28.23 ± 12.72	0.218
Estradiol on trigger day (pmol/l)	4337.26 ± 2130.30	4718.70 ± 2338.80	0.173
Mature oocytes (MII) retrieved	8.68 ± 5.01	9.26 ± 5.30	0.366
Fertilized oocytes (2PN)	7.86 ± 4.90	7.84 ± 5.07	0.977
No. of cleavage stage embryos	6.07 ± 4.16	5.86 ± 4.36	0.686
No. of blastocysts	3.05 ± 2.82	2.84 ± 2.23	0.521
No. of euploid blastocysts	1.72 ± 1.78		
No. of aneuploid blastocysts	1.28 ± 1.39		
No. of mosaic blastocysts	0.05 ± 0.22		

RPL = recurrent pregnancy loss; BMI = body mass index; AMH = anti-Müllerian hormone; FSH = follicle stimulating hormone; AFC = antral follicle count

#### SXCI status and AR CAG polymorphism of patients in the RPL and control groups

**Fig. 2:**
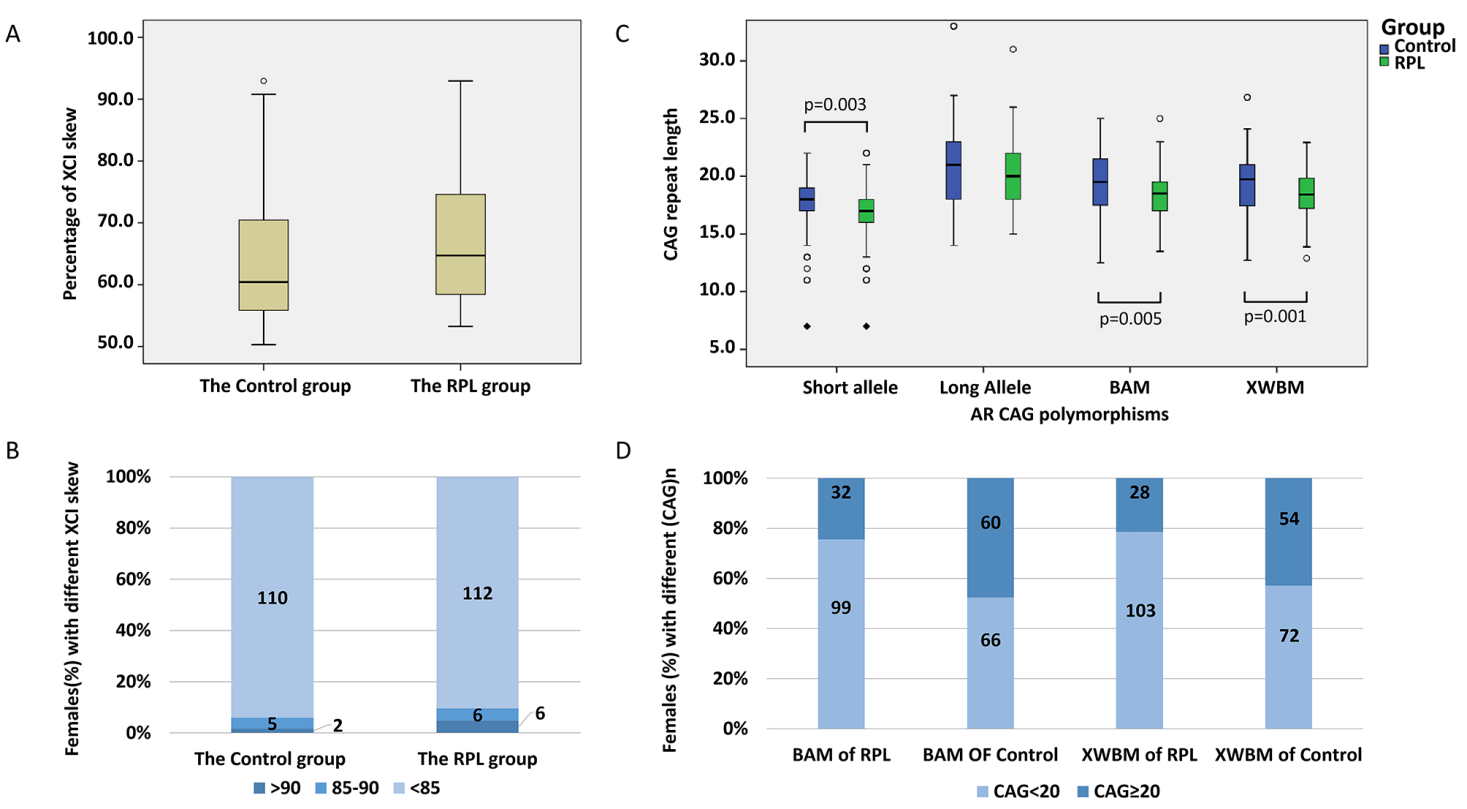
** A) Comparison of the XCI skew between the RPL and control groups**. The XCI skew is significantly higher in patients with RPL than in the controls (mean ± standard deviation, 67.71 ± 10.50 vs. 64.22 ± 10.62, p = 0.011). XCI = X-chromosome inactivation; RPL = recurrent pregnancy loss **B) Comparison of the AR CAG repeat polymorphism between the RPL and control groups.** The short allele (16.87 ± 2.39, vs. 17.77 ± 2.37, p = 0.003) and BAM (18.56 ± 1.97 vs. 19.34 ± 2.38, p = 0.005) were significantly shorter in patients with RPL than in controls. The difference in bioactive CAG repeats (XWBM) was more pronounced (18.46 ± 2.02 vs. 19.38 ± 2.53, p = 0.001). AR CAG = androgen receptor CAG; RPL = recurrent pregnancy loss; XWBM = X-weighted-biallelic-mean. **C) Percentage of women with XCI skew > 90, 85–90, and < 85 in the RPL and control groups.** The prevalence of highly skewed XCI (≥ 85%) and extremely skewed XCI (≥ 90%) were not significantly different between the two groups. XCI = X-chromosome inactivation; RPL = recurrent pregnancy loss **D) Comparison of patients with RPL and controls according to CAG repeats.** The median CAG repeat length of the control group was 19.50 or 19.72 calculated as BAM or XWBM, such that 20 was used as the cut-off value. The distribution was significantly different between the two groups (chi-square test, p-value = 0.000 for BAM, p-value = 0.000 for XWBM). RPL = recurrent pregnancy loss; XWBM = X-weighted biallelic mean; BAM = biallelic mean.

The AR gene showed heterozygosity rates of 94.6% and 92.8% in the RPL and control groups, respectively. The distribution of AR CAG repeat polymorphisms were within HWE (control group, p = 0.596; RPL group, p = 0.558). The distribution of XCI demonstrated a significantly more skewed pattern in patients with RPL than in controls (Fig. 2 A). The prevalence of highly (SXCI > 85%) or extremely (SXCI > 90%) skewed XCI was higher in the RPL group than in the control group; however, the difference was not significant (Fig. 2B). The short allele, the BAM, and XWBM of the AR CAG repeats were significantly shorter in patients with RPL than in controls (Fig. 2 C). The distribution of patients with RPL and controls according to the length of CAG repeats differed significantly (Fig. 2D).

The multivariate logistic regression analysis revealed that CAG repeat < 20, SXCI, and duration of stimulation were independently associated with RPL (Table 2).

**Table 2:** Logistic regression models

Variables	P-value	Adjusted OR/RR^a^	95%CI
Characteristics in relation to RPL*			
CAG < 20	0.000	3.012	1.648–5.504
SXCI	0.002	63.064	4.381-907.785
Duration of stimulation	0.022	0.866	0.798–0.983
Characteristics in relation to DOR**			
**RPL group (participants in the case–control study, N = 131)**			
Age	0.000	1.891	1.341–2.665
AMH	0.001	0.214	0.090–0.511
**Control group (N = 126)**			
CAG < 20	0.039	15.384	1.148-204.904
Age	0.005	1.622	1.154–2.280
AMH	0.001	0.026	0.003–0.210
**RPL cohort (participants in the external validation cohort, N = 363)**			
Age	0.001	1.295	1.116–1.502
AMH	0.000	0.104	0.045–0.237
AFC	0.036	0.905	0.824–0.993
Characteristics in relation to blastocyst euploidy in patients with RPL***			
**RPL group (participants in the case–control study, N = 131)**			
AFC	0.000	1.336	1.164–1.532
**RPL cohort (participants in the external validation cohort, N = 363)**			
Age	0.003	0.916	0.864–0.970
AFC	0.000	1.189	1.122–1.261

^a^Confounders are evaluated using multivariate logistic regression models (backward LR) and covariates are retained in the final adjusted model if they are significantly associated with the outcome parameters (p < 0.05)

*OR adjusted for age, AMH, BMI, short CAG allele, BAM and XWBM.

**RR adjusted for SXCI, CAG20, XWBM, age, AMH, AFC, and duration of stimulation

***RR adjusted for age, AFC, AMH, SXCI, CAG20, XWBM, BMI, number of pregnancies loss, and duration of stimulation

CI = confidence interval; OR = odds ratio; RR = risk ratio; RPL = recurrent pregnancy loss; SXCI = skewed X chromosome inactivation; XWBM = X-weighted biallelic mean; BMI = body mass index; AMH = anti-Müllerian hormone; AFC = antral follicle count

#### Association of SXCI and AR CAG polymorphisms with ovarian reserve

In total, 25 and 27 patients in the RPL and control groups were identified as having DOR, respectively. The DOR incidence was similar between the two groups (P = 0.640). The multivariate logistic regression analysis indicated CAG < 20 was independently associated with DOR in the control group, while SXCI and AR CAG polymorphisms were not associated with DOR in the RPL group (Table 2).

#### Association of SXCI and XWBM with blastocyst aneuploidy

The PGT-A results in the RPL group indicated that 95 patients acquired euploid blastocysts, whereas 36 patients did not. The logistic regression analysis revealed that neither SXCI nor AR CAG polymorphisms was related to blastocyst aneuploidy, whereas AFC was identified as the only independent factor affecting aneuploidy after adjusting for confounders (Table 2).

### External validation in the separate RPL cohort

To validate the associations of SXCI and AR CAG polymorphisms with DOR and blastocyst aneuploidy, we recruited a separate RPL cohort of 363 patients with similar baseline and cycle characteristics, SXCI status, and AR CAG polymorphisms compared with the patients with RPL in the case–control study (Table 3). In the validation cohort, 73 patients were identified as DOR, and 98 patients failed to acquire euploid blastocysts. The multivariate logistic regression analysis indicated that SXCI and AR CAG polymorphisms were not associated with the ovarian reserve or blastocyst aneuploidy, which was in accordance with the results revealed in the RPL group from the case–control study (Table 2).

**Table 3:** Characteristics of patients with RPL in the external validation cohort

	RPL cohort	P-value*
	**N = 363**	
Age (years)	33.58 ± 4.64	NS
BMI	22.33 ± 1.48	NS
No. of pregnancy losses	3.21 ± 0.71	NS
E2 (pg/ml)	40.45 ± 20.05	NS
T (ng/dL)	44.69 ± 17.36	NS
**Ovarian reserve**		
AMH	3.96 ± 3.60	NS
FSH on day 2	7.85 ± 2.63	NS
AFC	11.64 ± 6.85	NS
**Cycle characteristics**		
Duration of stimulation	9.42 ± 2.48	NS
Total dosage of gonadotrophins	26.82 ± 11.59	NS
Estradiol on trigger day (pmol/l)	4236.62 ± 2270.31	NS
Mature oocytes (MII) retrieved	9.38 ± 6.09	NS
Fertilized oocytes (2PN)	8.36 ± 5.74	NS
No. of cleavage stage embryos	6.48 ± 4.64	NS
No. of blastocysts	3.11 ± 2.89	NS
No. of euploid blastocysts	1.86 ± 1.92	NS
No. of aneuploid blastocysts	1.31 ± 1.42	NS
No. of mosaic blastocysts	0.06 ± 0.23	NS

*Comparison with patients in the RPL group

## Discussion

In this study, patients with RPL demonstrated a significantly more skewed SXCI distribution pattern and significantly shorter BAM and XWBM of AR CAG repeats than controls. However, SXCI and AR CAG polymorphisms were not associated with ovarian reserve or preimplantation embryo aneuploidy. These findings indicate that the role of XCI and AR CAG polymorphisms in RPL may not be associated with embryonic aneuploidy.

Evidence of the association between SXCI and RPL is conflicting [29, 39–42]. A previous meta-analysis has demonstrated that extreme XCI skewing (≥ 90%) is associated with idiopathic RPL with ≥ 3 pregnancy losses, while the significance diminished when RPL was defined as ≥ 2 losses or SXCI was defined as ≥ 85% [13].In the present study, we observed a significantly more skewed distribution pattern of XCI in patients with RPL than in controls. Meanwhile, the present study for the first time revealed SXCI is independently associated with RPL using multivariate logistic regression model after adjusting for possible confounders.

Several groups [27, 28, 43, 44] have reported a relationship between AR gene polymorphisms and RPL, with the results being inconsistent. Aruna et al. demonstrated that longer CAG repeat lengths are associated with increased odds for RPL in Indian women [27]. Conversely, Chuan et al. demonstrated that shorter CAG repeat lengths are associated with an increased risk of RPL [28]. In the present study, significantly shorter AR CAG was observed in patients with RPL than in controls, and the difference was greater when the active X chromosome was considered. These results are consistent with the results obtained by Chuan et al., who also conducted their study in Chinese women [28]. Therefore, we inferred that ethnic differences may be at play although this could also be a stochastic event due to the small sample size and more studies are needed to elucidate clear relationships between AR gene polymorphisms and RPL in different races. Considering the inverse effect of CAG repeat length on receptor activity, alleles with shorter CAG repeat lengths are expected to amplify AR activity. Although serum testosterone levels were similar in the RPL and control groups (Table 1), we hypothesized that AR CAG repeat polymorphisms might cause different effects in the downstream of the receptor and play a role in RPL.

In addition to more skewed XCI and shorter CAG repeats, women with RPL exhibited a shorter stimulation duration than did the controls despite receiving the same antagonist protocol with similar total gonadotropin dosages. Recent studies have reported that a shorter follicular phase length is associated with DOR and poorer oocyte quality [45–47] and as such, women with RPL might have undergone a potential decrease in ovarian reserve, although the incidence of DOR, which was defined by AMH and AFC, remained similar compared with the controls. We investigated the relationships between SXCI, CAG repeats, and stimulation duration, and observed that neither SXCI nor XWBM was significantly associated with the duration of stimulation in the RPL group or in the control group. This is consistent with the results of a study by Lledó et al., who showed that CAG repeat length was not associated with stimulation length in a population of fertile egg donors [48].

DOR and embryo aneuploidy are associated with RPL. A recent meta-analysis by Bunnewell et al. has revealed that low AMH and AFC levels were predictive of the higher odds for RPL [34]. In addition, fetal aneuploidy accounts for approximately 50% of pregnancy losses [49] and PGT-A improved live birth rates in couples with RPL undergoing frozen embryo transfer [8]. Therefore, we inferred that SXCI and AR CAG polymorphisms might influence ovarian reserve or embryo aneuploidy and therefore, cause RPL. However, SXCI, XWBM, and CAG ≥ 20 were not associated with the ovarian reserve or blastocyst aneuploidy in our study after adjusting for confounders in RPL patients, suggesting that SXCI and AR CAG play a role in RPL through other mechanisms.

In previous studies, XCI patterns and AR CAG polymorphisms were demonstrated to influence the etiopathogenesis of DOR, even though the results were conflicting. A meta-analysis by Pu et al., involving 325 cases and 403 controls, showed that skewed XCI was not associated with premature ovarian failure (POF) [50]. However, a recent study by Miranda-Furtado et al. demonstrated a significantly higher frequency of skewed XCI in women with idiopathic premature ovarian insufficiency than in controls [14]. Sugawa et al. [51] and Laisk et al. [52] have revealed that CAG repeat lengths were significantly shorter in patients with POF than in healthy women. However, the results of the studies by Lledó et al. [53] and Chatterjee et al. [54] suggested that women with POF had longer CAG repeat lengths than the controls. Nevertheless, Panda et al. observed no significant differences between women with POF and healthy controls [55]. In the present study, shorter CAG repeats was significantly associated with DOR in the control group, which was consistent with the results of Sugawa et al. [51] and Laisk et al. [52]. However, neither SXCI nor AR CAG repeat length interfered with DOR in patients with RPL, and the results were validated in an external cohort with sufficient sample size.We believe the variant results among existing studies might be related to small sample size or ethnic differences.

To the best of our knowledge, this is the first study to investigate the correlation between SXCI and CAG polymorphisms with embryo euploid status at the preimplantation blastocyst stage. Previously, only three studies have investigated the association between SXCI and embryo aneuploidy, and all have examined the products of conception. Sangha et al. demonstrated that RPL patients with SXCI > 90% had more miscarriages secondary to fetal aneuploidy than those without extreme SXCI [30]. Beever et al. demonstrated that the proportion of women with SXCI > 90% was significantly higher in the group with pregnancy loss secondary to trisomy of proven maternal meiotic origin than in the control group [31]. However, Warburton et al. observed no significant difference in highly skewed SXCI (> 85%) between the group of women with trisomic pregnancy losses and the age-matched fertile women, whereas a significantly increased prevalence of SXCI was observed in the group of women with non-trisomy aneuploid pregnancy losses than in the controls [32]. Our results did not support an association between blastocyst aneuploidy and SXCI or AR CAG polymorphisms in patients with RPL, indicating that patients with RPL with SXCI or short AR CAG repeats are not at a higher risk of blastocyst aneuploidy than those without SXCI or those within the normal range of CAG repeats.

The limitations of the present study need to be addressed. First, the sample size of the case-control study is limited considering the overall size of the Chinese population and the prevalence of RPL. Despite that we enrolled a separate RPL cohort with statistically sufficient sample size to validate the results and that we adjusted many potential confounders by logistic regressions, the potential confounding factors cannot be entirely ruled out. Second, this study was carried out in the Chinese Han population, while the race and ethnicity differences may have an effect on the genetic polymorphisms, such that further research are needed to estimate the role of SXCI and AR CAG repeat polymorphisms in RPL risk within other populations.

According to the above results, we concluded that mechanisms other than ovarian reserve or embryo aneuploidy may account for this association of SXCI and AR CAG polymorphisms with RPL. Chromosome X is also enriched for immune-related genes, and skewed XCI patterns could cause the breakdown of thymic tolerance induction processes, conferring an increased predisposition to develop autoimmunity [12], which might lead to abnormal immune responses at the maternal–fetal interface and result in pregnancy loss. Further studies with larger sample sizes and in different races are needed to confirm our results and to explore the effects of SXCI and AR CAG polymorphisms on immunity in patients with RPL.

## Conclusion

In summary, SXCI and AR CAG polymorphisms are related to RPL; however, these two factors do not cause RPL by affecting the ovarian reserve and increasing embryo aneuploidy. The roles of SXCI and AR CAG in RPL may involve other mechanisms that require further investigation.

## Data Availability

Data of AR CAG repeat polymorphisms of the case-control study are deposited in the Science Data Bank (SCIDB database, accession link: https://www.scidb.cn/en/detail?dataSetId=ddbbf433c4dd4b2cb2af55c9a40b66f9; DOI:10.57760/sciencedb.02147) and National Genomics Data Center-OMIX database (Project No. PRJCA010969, accession link: https://download.cncb.ac.cn/OMIX/OMIX001520/).

## Abbreviations

CI: confidence interval
RR: risk ratio
RPL: recurrent pregnancy loss
SXCI: skewed X chromosome inactivation
XWBM: X-weighted biallelic mean
BMI: body mass index
AMH: anti-Müllerian hormone
AFC: antral follicle count
AR: androgen receptor
PGT-A: preimplantation genetic testing for aneuploidy
FSH: follicle-stimulating hormone
DOR: diminished ovarian reserve
